# Influence of phytocenosis on the medical potential of moss extracts: the *Pleurozium schreberi* (Willd. ex Brid.) Mitt. case

**DOI:** 10.1038/s41598-023-47654-z

**Published:** 2023-11-20

**Authors:** Grzegorz J. Wolski, Agnieszka Kobylińska, Beata Sadowska, Anna Podsędek, Dominika Kajszczak, Marek Fol

**Affiliations:** 1 Department of Geobotany and Plant Ecology, Faculty of Biology and Environmental Protection, University of Lodz, Ul. Banacha 12/16, 90-237 Lodz, Poland; 2 Department of Plant Ecophysiology, Faculty of Biology and Environmental Protection, University of Lodz, Ul. Banacha 12/16, 90-237 Lodz, Poland; 3 Department of Immunology and Infectious Biology, Institute of Microbiology, Biotechnology and Immunology, Faculty of Biology and Environmental Protection, University of Lodz, Ul. Banacha 12/16, 90-237 Lodz, Poland; 4 Institute of Molecular and Industrial Biotechnology, Faculty of Biotechnology and Food Sciences, Lodz University of Technology, Ul. Stefanowskiego 2/22, 90-537 Lodz, Poland

**Keywords:** Plant sciences, Medical research

## Abstract

The question was asked “whether plant phytocenosis has an impact on the medical potential of the extracts from *Pleurozium schreberi*”. Moss samples were collected from four different phytocoenoses: mixed forest (oak-pine forest), a forest tract in pine forest, 5–15-year-old pine forest and 50-year-old pine forest. Chemical composition of the extracts, antioxidative capacity (FRAP and ABTS^·+^ assays), as well as biological activities including cytotoxicity for the mouse fibroblasts L929 line (MTT reduction assay), biostatic/biocidal effect against selected bacteria and fungi (broth microdilution method followed by culture on solid media), and regenerative properties on human fibroblasts HFF-1 line (scratch assay) were tested. The conducted research clearly proves that phytocenosis determines the quality of moss extracts. The analyses showed that in every examined aspect the IV-7 extract (obtained from a specimen collected in a *Pinus sylvestris* L. forest, monoculture up to 15 years old) exhibited the highest values and the strongest activity. Other extracts of the same species but growing in other phytocenoses—in a mixed forest (IV-5), a forest tract in a *Pinus sylvestris* monoculture forest (IV-6) and in a *P*. *sylvestris* forest of pine monoculture about 50 years old (IV-8) showed much weaker activity and lower values of the above-mentioned parameters. At the same time, none of the tested extracts exerted a pro-regenerative effect. The *P. schreberi* extracts were characterized by a varied total content of phenolic compounds in the range from 0.63 ± 0.02 to 14.01 ± 0.25 mg/g of plant material. UPLC/MS analysis showed a varied phenolic profile of the extracts, with caffeoylquinic acid and quercetin triglucoside predominating in all of them.

## Introduction

Bryophytes are quite rarely examined in terms of their medical potential^1^. More common objects of research are vascular plants, even those coming from different parts of the world, e.g. from the tropics^2–5^. However, as some studies show, highly biologically active extracts can be also obtained from plants growing in central Europe^1^.

Among bryophytes, only liverworts have a long tradition of being studied in medical terms. This research has been carried out systematically for many years^6–10^. On the other hand, mosses seem to be analyzed in this respect rather selectively and quite accidentally^11–22^.

The analysis is usually focused only on the antioxidant potential^11–22^. In most cases the researchers choose large, easily recognizable species of mosses, generally from forests^11,17,22,23^, and much less frequently taxa of other ecosystems^1,16,18,19^. Thereby, the obtained results sometimes make their full interpretation impossible. It is hard to state whether they are typical of the species, genus, habitat or phytocenosis conditions in which a given taxon develops, or they are more random and dependent on completely different factors—season, temperature, precipitation.

Until now few selected compounds, including antioxidants have been studied^24–26^. Wolski et al.^1^ examined species from various substrates (e.g. epigeites, epillites, epiphytes) of the urban ecosystem, indicating that some of them are characterized by high levels of antioxidants and high medical potential. However, it is impossible to conclude on this basis which has a greater impact on the level of the medical potential of these plants—genus, substrate or habitat conditions. There is no data which would allow comparing the role of the habitat, microhabitat, substrate, ecological conditions or the impact of the phytocenosison the concentration of biologically active substances in the tissues of the taxa studied^24–27^.

Considering the above facts, we wanted to shed some light on the influence of phytocenosis conditions on the medical potential of moss tissue extracts. Thus, the primary purpose of the following studies was to investigate the impact of the phytocenosis in which *Pleurozium schreberi* Willd. ex Brid.) Mitt. was collected on the medical potential of its extracts. Secondary objectives were to determine the total phenolic content; assess the chlorophyll concentration; test antioxidant capacity; quantify cytotoxicity; verify antimicrobial activity; and determine pro-regenerative activity of the analyzed extracts.

We hypothesized that the phytocenosis in which *P*. *schreberi* grow affects the parameters of the extracts.

## Results

### General results

The conducted research showed that the tested extracts were qualitatively different. The analysis of the material clearly revealed that phytocenosis determined the medical potential of the extracts of the studied species. Phytocenosisis characterized by specific parameters (Table 1) including light intensity. The extracts differed in terms of almost all analyzed factors, from *P*. *schreberi* extracts collected in a *Pinus sylvestris* forest (monoculture up to 15 years old) showing the highest phenolic compounds content values; power of ferric ion reduction and radical scavenging activity as well as the highest decrease in L929 cell survival and the highest fungistatic and fungicidal activity. Importantly, this phytocenosis was also characterized by the highest light intensity (Table 1). The remaining extracts exhibited significantly lower values of the above-mentioned factors.

**Table 1:** Researched phytocenosis and their selected parameters.

The sample number	IV-5	IV-6	IV-7	IV-8
Description of the phytocenosis	Mixed forest (oak-pine forest)	A forest tract in pine forest	Pine forest 5–15 yearsold	Pine forest 50 yearsold
GPScoordinates	51° 25′ 58″ N	51° 31′ 48″ N	51° 26′ 32″ N	51° 27′ 07″ N
	20° 01′ 29″ E	19° 58′ 22″ E	19° 46′ 30″ E	19° 49′ 21″ E
Klx[kg lux]	0.03–0.06	0.05	0.1	0.02

### Content of phenolic compounds

Phenolic compounds are important plant constituents with the redox properties responsible for antioxidant activity. The results of the colorimetric analysis of the total phenolic content (TPCs) based on the absorbance of the extract solutions reacting with the Folin-Ciocâlteu reagent (FCR), expressed as mg gallic acid equivalent (GAE) per 1 g DW of extract, are given in (RawDate 1). *Pleurozium schreberi* were taken from the different analyzed areas. The highest phenolic content was found in IV-7, and significantly lower TPCs (by about 40%)—18.9 ± 0.9 and 17.1 ± 1.4 mg GAE/1 g DW of extract—were determined for IV-8 and IV-5, respectively. Extract IV-6 obtained from the same species but taken from a different phytocenosis had an approximately 60% lower phenolic level in comparison to the IV-7 sample.

The results of the qualitative and quantitative analysis of mosses obtained by the UPLC/MS method are presented in Tables 2 and 3. Individual phenolic compounds were identified based on retention times (Rt), Uv–Vis spectra, deprotonated molecules ([M-H]), diagnostic fragments (MS/MS) and comparison with the standard reference compounds (chlorogenic acid and cryptochlorogenic acid). The tentative identification of the seven compounds was based on the literature data (Table 2). The sum of the identified phenolic components varied from 1.10 ± 0.01 to 2.60 ± 0.05 mg/g DW of extract (Table 3). Quantitatively, in the group of identified phenolic compounds, quercetin triglucoside and caffeoylquinic acid dominated in extracts IV-5, IV-6, IV-7and IV-8.

**Table 2:** Profile of phenolic compounds in the extracts obtained by UPLC/MS analysis.

No.	R_t_ (min)	λ_max_ (nm)	${[M-H]}^{-}$ (m/z)	MS/MS (m/z)	Compound	References
1	3.99	322	353	119,182,128	Chlorogenic acid	Standard
2	4.14–4.36	323	353	183,153,255,136	Cryptochlorogenic acid	Standard
3	5.20–5.64	313	353	191,249, 292,221	Caffeoylquinic acid	^28^
4	6.70–7.16	336	769	269,225,299,117	Quercetin triglucoside	^29^
5	11.39–11.86	320	325	187,119,309, 163	*p*-Coumaroylhexoside I	^30^
6	11.56–12.03	320	337	183,119,191	Coumaroylquinic acid I	^31^
7	11.61–11.94	320	325	187,119,309, 163	*p*-Coumaroylhexoside II	^30^
8	12.04–12.30	320	337	183,119,191	Coumaroylquinic acid II	^31^
9	12.67–12.71	328	381	179,123,119	Caffeoylquinic acid derivative I	^30^

**Table 3:** Content of identified phenolic compounds in the extracts.

Compound	IV-5	IV-6	IV-7	IV-8
	µg/g			
Chlorogenic acid	–	–	–	395 ± 7
Cryptochlorogenic acid	–	–	45 ± 3a	110 ± 4b
Caffeoylquinic acid^1^	320 ± 17b	982 ± 7d	284 ± 3a	733 ± 4c
Quercetin triglucoside^2^	497 ± 16b	517 ± 20b	402 ± 1a	421 ± 20a
*p*-Coumaroylhexoside I^1^	211 ± 7b	–	–	126 ± 4a
Coumaroylquinic acid I^1^	–	–	95 ± 1a	222 ± 3b
*p*-CoumaroylhexosideII^1^	197 ± 10c	40 ± 1a	78 ± 2b	246 ± 9d
Coumaroylquinic acid II^1^	–	155 ± 7b	82 ± 1a	352 ± 3c
Caffeoylquinic acid derivative I^1^	272 ± 3c	195 ± 5b	118 ± 1a	–

“–”, not detected. Results are expressed as a mean ± standard deviation (n = 3). Different letter in each row represent significant differences based on Tukey’s post-hoc test at p < 0.05. The content expressed as equivalents of: ^1^chlorogenic acid; ^2^quercetin 3-glucoside.

### Content of chlorophyll pigments

Chlorophyll concentrations in the analyzed moss extracts were very diverse (Table 4). Chlorophyll *a* contents ranged from 0.40 to 7.77 mg/g while those of chlorophyll *b* were from 0.49 to 4.70 mg/g. Samples IV-6 were characterized by the lowest content of both chlorophyll forms. The chlorophyll *a* content was higher than that of chlorophyll *b *in IV-5 and IV-7, but in samples IV-6 and IV-8 chlorophyll *b* dominated.

**Table 4:** Content (mg/g) of chlorophyll pigments in the extract.

Sample	Chlorophyll *a*	Chlorophyll *b*	Chlorophyll *a* + *b*	Ratio of chlorophylls *a*/*b*
IV-5	2.87	1.59	4.46	1.82
IV-6	0.40	0.49	0.89	0.82
IV-7	7.77	4.70	12.47	1.65
IV-8	1.44	2.81	4.25	0.51

Results are expressed as a mean (n = 2).

### Antioxidant capacity

Our data indicated that both radical scavenging activity and reducing properties in the analyzed extracts decreased in the following order: IV-7, IV-8, IV-5, IV-6. The trolox equivalent for extract IV-7 was only slightly (10%) higher than that for IV-8 and about 30% higher in comparison to extract IV-5. The lowest antioxidant capacity was obtained for extract IV-6 and it was 12.3 ± 1.25 µM Trolox *vs*. 33.05 ± 3.46 µM Trolox for IV-7. A similar tendency was observed using the FRAP method. The ferric ion reducing antioxidant power determined for extract IV-7 compared to IV-8, IV-5 and IV-6 was stronger by about 30%, 35% and 50%, respectively (RawDate 3).

### Cell viability in the presence of moss extracts: cytotoxicity quantification

**Figure 1:**
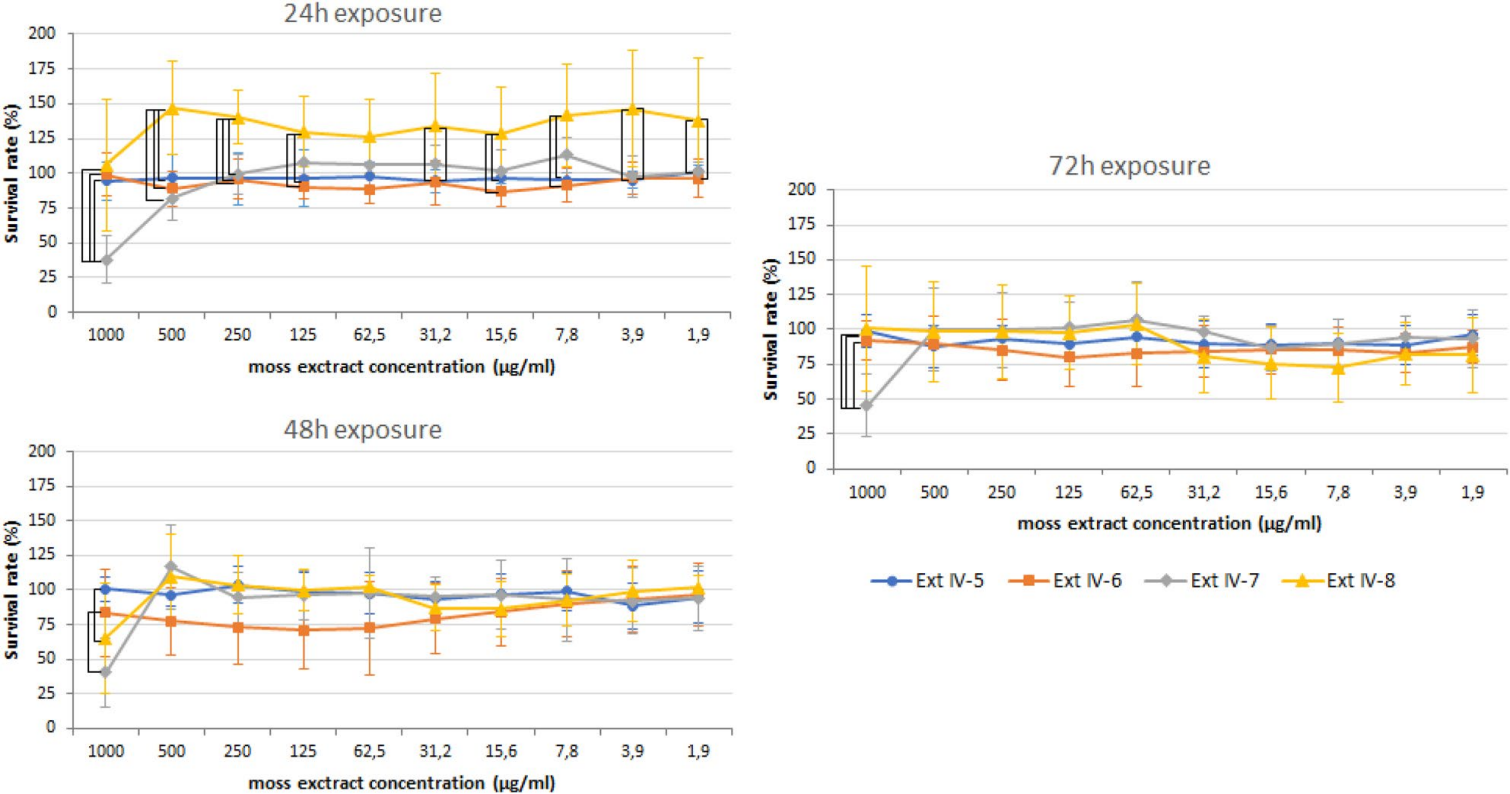
Dose dependent effects of ethanolic *P. schreberi* extracts (IV-5, IV-6, IV-7, IV-8) on L929 cell viability after 24, 48 and 78 h of exposure. The survival rate was quantified using the MTT colorimetric method. The results are expressed as median ± SD. Statistical analyses were performed with the STATISTICA 12.0 PL software. Differences between the samples were analyzed using the variance Kruskal–Wallis non-parametric test. Statistical differences were marked with the square bracket. P-values of ≤ 0.05 were considered significant.

A 24-h culture of L929 cells with extract IV-8 did not cause a cytotoxic effect in the tested concentration range (Fig. 1). On the contrary, the survival values, markedly higher compared to those obtained after using other extracts (although not statistically significant), may suggest that extract IV-8 could have positively affected cell proliferation. Moreover, prolonged incubation of fibroblasts with extract IV-8 did not cause a drastic decrease in the survival of L929 cells (after 48 h—close to 100% except at the highest concentration, and after 72 h—around 75%) suggesting the absence of cytotoxic properties of this extract.

Extract IV-7 at a concentration of 1000 µg/ml caused a drastic decrease in L929 cell survival to about 40% after a 24-h exposure, and this effect persisted also when the incubation was prolonged to 48 and 72 h. No cytotoxic effect was observed at the other concentrations of extract IV-7 (Fig. 1).

The survival rate of L929 cells after a 24-h exposure to extract IV-6 indicated no or very little cytotoxic effect, as the percentage of dead cells did not exceed 25% at any of the tested concentrations (Fig. 1). Under these conditions, the concentrations of the extract fell within or barely exceeded the so-called first toxic dose (up to 10%). Furthermore, the fibroblast exposure to the extract, even when prolonged up to 48 or 72 h did not result in the deaths of 50% or more cells, and thus extract IV-6 did not achieve the so-called measure of cytotoxicity in the tested concentration range.

Extract IV-5 showed no cytotoxic effect , and even when the exposure of the cells was prolonged to 72 h, it did not lead to such an effect (the decrease in thesurvival rate did not exceed 20%).

### Antimicrobial activity

The antimicrobial activity of *P. schreberi* extracts (IV-5, IV-6, IV-7, IV-8) and selected antibiotics (oxacillin (Oxa), gentamycin (Gen), and fluconazole (Flu)) against reference bacterial and yeast strains are presented in Table 5. as a minimum inhibitory concentration (MIC) and a minimum bactericidal/fungicidal concentration (MBC/MFC). The *P. schreberi* extracts did not exhibit biostatic or biocidal activity at the whole concentration range tested (up to 2000 µg/mL). Only the IV-7 extract showed fungistatic and fungicidal activity against *Candida glabrata* ATCC 90030 with MIC and MFC at the level of 1000 µg/mL. However, such an effect was not observed against *C*. *albicans*.

**Table 5:** The antimicrobial activity of *P*. *schreberi* extracts and selected antibiotics.

Microorganism	MIC [µg/mL] MBC/MFC [µg/mL]				
	IV-5	IV-6	IV-7	IV-8	Antibiotic
Bacteria					
* Staphylococcus aureus* ATCC 29213	> 2000	> 2000	> 2000	> 2000	0.125
	> 2000	> 2000	> 2000	> 2000	1
* Staphylococcus aureus* ATCC 43300	> 2000	> 2000	> 2000	> 2000	> 2
	> 2000	> 2000	> 2000	> 2000	> 2
* Staphylococcus epidermidis* ATCC 12228	> 2000	> 2000	> 2000	> 2000	0.25
	> 2000	> 2000	> 2000	> 2000	0.5
* Enterococcus faecalis* ATCC 29212	> 2000	> 2000	> 2000	> 2000	8
	> 2000	> 2000	> 2000	> 2000	32
* Escherichia coli* ATCC 25922	> 2000	> 2000	> 2000	> 2000	1
	> 2000	> 2000	> 2000	> 2000	2
* Pseudomonas aeruginosa* ATCC 25619	> 2000	> 2000	> 2000	> 2000	1
	> 2000	> 2000	> 2000	> 2000	4
Fungi					
* Candida albicans* ATCC 10231	> 2000	> 2000	> 2000	> 2000	> 64
	> 2000	> 2000	> 2000	> 2000	> 64
* Candida glabrata* ATCC 90030	> 2000	> 2000	1000	> 2000	64
	> 2000	> 2000	1000	> 2000	> 64

MIC, minimum inhibitory concentration measured by broth microdilution method; MBC/MFC, minimum bactericidal/fungicidal concentration determined based on a microbial culture on solid media; Antibiotic, oxacillin (Oxa) against staphylococci, gentamycin (Gen) against the other bacteria, and fluconazole (Flu) against fungi.

### The effect of *P. schreberi* extracts on regenerative properties of fibroblasts in a scratch assay

**Figure 2:**
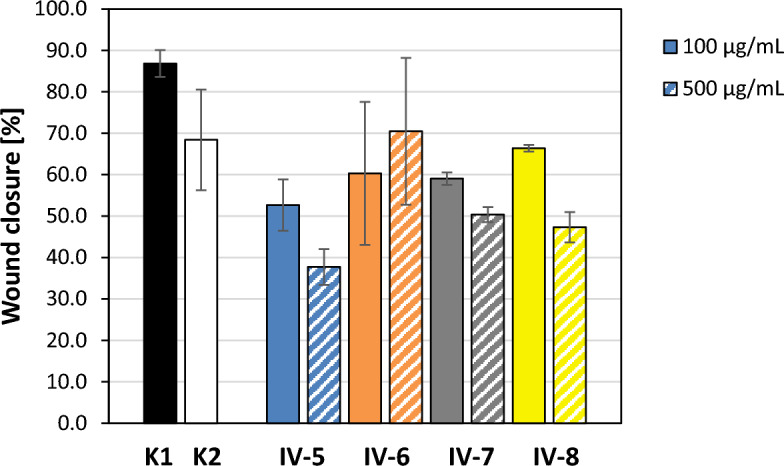
Wound closure tested in vitro by the scratch assay on human foreskin fibroblasts (HFF-1 line) in the presence of *P*. *schreberi* extracts.

**Figure 3:**
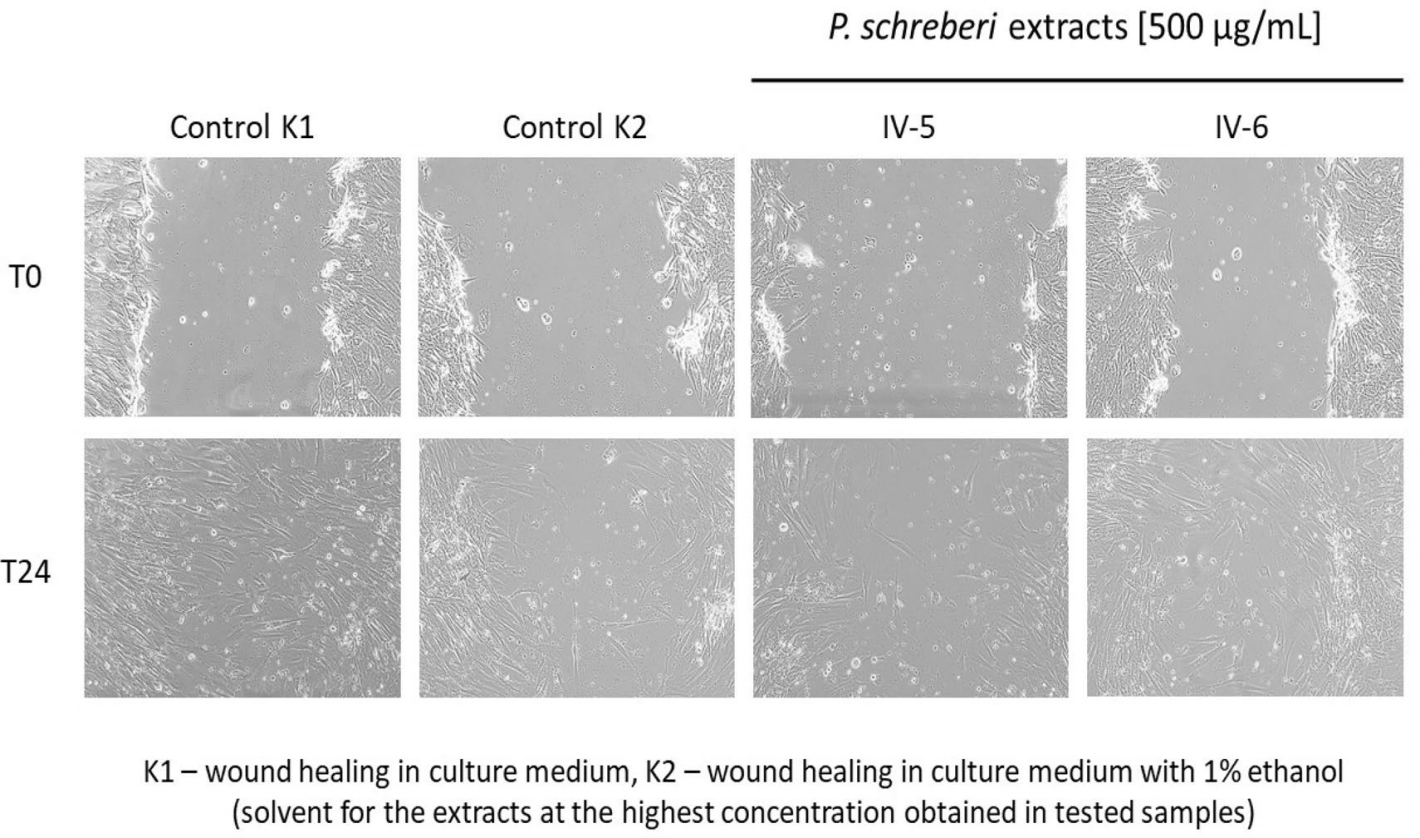
The effect of selected *P*. *schreberi* extracts on human foreskin fibroblasts (HFF-1 line) migration tested in vitro by the scratch assay. Representative images of the prepared wound just after scratching (T0) and after a 24 h exposure to the extracts (T24), magnification: 10 × 20.

The in vitro scratch assay on the human fibroblasts HFF-1 line was used to test a possible pro-regenerative activity of *P*. *schreberi* extracts. The extracts were used at two non-cytotoxic concentrations: 100 and 500 µg/mL. The obtained results are presented in Table 6, Figs. 2 and 3. The presence of ethanol at a concentration of 1% in the culture medium (control K2) inhibited the rate of wound healing (decrease of 18.42% on average) compared to the wound healing in the culture medium alone (control K1). Therefore, the results for the cells exposed to *P*. *schreberi* extracts used at a concentration of 500 µg/mL (the final ethanol concentration in those samples was 1%) should be referred to the results of K2, while the results for the cells exposed to the extracts used at 100 µg/mL (the final ethanol concentration in those samples was 0.2%) could be referred to the results of K1. All of the tested extracts weakened HFF-1 migration and wound healing compared to appropriate controls, thus none of them showed pro-regenerative activity. The strongest inhibition was noted for the IV-5 extract (by 34.14% and 30.68% on average, when used at 100 µg/mL and 500 µg/mL, respectively).

**Table 6:** The effect of *P*. *schreberi* extracts on human foreskin fibroblasts (HFF-1 line) migration tested in vitro by the scratch assay.

Sample	Extract concentration [µg/mL]	Average wound area at T0 [pixels^2^ ± SD]	Average wound area at T24 [pixels^2^ ± SD]	Average wound closure [% ± SD]
IV-5	100	2,066,524 ± 613,268	996,956 ± 418,165	52.68 ± 6.19
	500	1,973,597 ± 579,405	1,216,525 ± 275,197	37.72 ± 4.34
IV-6	100	2,165,320 ± 451,155	898,218 ± 552,633	60.32 ± 17.25
	500	1,984,384 ± 637,399	642,663 ± 540,132	70.46 ± 17.73
IV-7	100	1,985,880 ± 52,294	812,910 ± 8671	59.05 ± 1.52
	500	1,833,209 ± 37,513	909,579 ± 14,593	50.36 ± 1.81
IV-8	100	2,018,906 ± 361,615	679,913 ± 137,854	66.40 ± 0.81
	500	2,029,217 ± 523,438	1,059,461 ± 201,029	47.31 ± 3.68
Control K1	–	1,682,782 ± 174,903	225,596 ± 77,101	86.82 ± 3.24
Control K2	–	1,821,926 ± 244,797	595,264 ± 304,190	68.40 ± 12.15

T0, T24, time [h] after wounding; K1, wound healing in culture medium; K2, wound healing in culture medium with 1% ethanol (solvent for the extracts at the highest concentration obtained in the tested samples); SD, standard deviation.

## Discussion

Secondary metabolites of plants, such as phenolic acids, flavonoids, tannins, cumarins, lignins, carotenoids or essential oils possess a broad range of biological activity, which may have beneficial pro-health effect^32–34^. Bryophytes can be also a source of some of these compounds. Anti-oxidative, anti-inflammatory, anti-platelet, anti-leukemic or antimicrobial activities have been reported for bryophyte extracts^35–37^. However, unambiguous indication of the bioactivity of the material from a specific plant or its parts seems to be one of the most challenging aspects of research, since the composition of material depends on many environmental factors influencing plant morphology, anatomy and physiology^32^. Most plants contain a large number of phenolic compounds, especially polyphenols which, from a functional point of view, have adaptive roles. Polyphenols have a similar function in moss tissues. Therefore, studies of these compounds will help to understand their responses to environmental conditions *i.a*. UV radiation, protection against oxidative stress, as well as functions of the main secondary metabolites in the overall metabolism^26^. Phenolic compounds, especially flavonoids, are important in UVB absorption, and many literature data indicate that bryophyte species respond to high UVB radiation by accumulation of protective substances^26,38,39^. The analysis of active compounds accumulated in moss tissues indicates changes in total phenolic content connected with seasons^40^. Moreover, correlation between the climatic parameters (sampling time, temperature and precipitation) and chemical characteristics of moss extracts has been found (Klavina et al.)^41^.

The results of colorimetric analysis of the total phenolic content in *P*. *schreberi* extracts showed that the mosses taken from different analyzed areas had significant differences in the level of phenolics. The highest phenolic content was found in extract IV-7 (pine forest 5–15 years old). About 40% lower amounts of phenolic substances were determined for IV-8 and IV-5 (pine forest 50 years old and mixed forest, respectively). The extract from the same species but taken from a forest tract in a pine forest had an approximately 60% lower phenolics level in comparison to extract IV-7. On the other hand, the research conducted by Klavina et al.^26^ showed a strong connection between the habitat and the total amount of phenolics. In the cited studies it was observed that the highest total phenolics content was observed for species growing in coniferous forests and humid habitats^26^. In contrast to this study, our results demonstrate that the highest level of these secondary metabolites was observed for samples taken from the sunniest phytocenosis—a 5–15 year-old pine forest.

Generally, it is accepted that most plants containing a large number of phenolic compounds exhibit antioxidant activity^17,42^. Moreover, the biosynthesis of effective antioxidant flavonoids is enhanced when plants grow under strong light^43^. Therefore, we checked our moss extracts toward their antioxidant potential. In order to determine total antioxidant capacity, ABTS and FRAP assays were used. The obtained results confirmed the data showing that the highest antioxidant power was reported for the samples having the highest phenolic content and exposed to the strongest sunlight. However, antioxidant activity is not always correlated with the amount of phenolic compounds. Our previous study demonstrated that among five analyzed moss species only in *Ceratodon purpureus* a positive correlation was found between the total content of phenolics and antioxidant capacity^1^. Chauke et al.^44^ pointed to a complication in the predictability of redox reactions in the mixture of compounds present in the cells of mosses because it is possible to expect both synergistic and antagonistic effects.

Analysis of the medical potential of moss extracts is not common in the literature. Usually, the research has been conducted in a single thread, most often studies have concerned the analysis of the content of phenolic compounds or generally antioxidants, much less often other aspects of tested extracts^11–22^ have been investigated.

In addition, studies conducted on bryophytes tend to focus on relatively large, easily identifiable forest species^11,13,14,17,22^. They are much less relevant to non-forest taxa associated with open ecosystems^1,12,14,15^.

In our previous research we focused on small specimens collected from the area of ​​a large city^1^. The results of these studies indicated that, compared to other medicinal plants, the antioxidant activity of the tested mosses could be considered high. Literature data indicate that the level of antioxidants is affected by the intensity of light radiation^43,45^. Currently conducted studies confirmed these data—the greatest antioxidant potential was exhibited by the extracts from *P. schreberi* tissues collected from a young pine phytocenosis (up to 15 years old), which was also characterized by the highest intensity of sunlight (Table 1).

*Pleurozium schreberi* has already been the subject of research in the context of medical potential, but studies on this species are not common in the literature^11,23,26^. In the reported analyses this taxon was compared only with other forest species. Due to the fact that many taxa of mosses have a very wide ecological amplitude—e.g. *P*. *schreberi*, it seems important to test the biological activity and medical potential of one species collected from different phytocenoses. The above-mentioned studies on *P*. *schreberi* are unfortunately difficult to compare with our results, because they do not contain information from which habitat or phytocenosis the material was collected^11,23^. Only Klavina et al.^41^described the phytocenosis of the studied taxa in detail, stating that the material was collected from a 60-year-old swamp forest.

Considering the potential medical use of natural products, it is necessary to evaluate their cytotoxicity against normal eukaryotic cells. To determine the potential cytotoxic effect of the tested moss extracts, we used the MTT method, which, despite some limitations, is characterized by several advantages that are difficult to challenge (simplicity, rapidity, sensitivity and repeatability), hence it is a tool widely used in biological research^46–50^. As described by Mosmann^51^, the concept behind this method is to measure spectrophotometrically the ability of living cells to enzymatically reduce the yellow water-soluble tetrazolium salt MTT [3-(4,5-dimethylthiazol-2-yl)-2,5-diphenyl tetrazolium bromide] by mitochondrial succinate dehydrogenase into a dark blue/purple water-insoluble formazan compound, whose amount is directly proportional to the number of live cells^52,53^. Although many cell lines, especially tumor cell lines, e.g. HeLa, HEP-G2, MCF-7, A431^54–57^, as well as the Vero cell line^58^ or even animal-derived splenocytes, thymocytes, hepatocytes^59^ are used for in vitro cytotoxic activity screening, the L929 line of mouse fibroblasts is one of the most widely used and recommended^60–64^. Plants produce bioactive compounds with a variety of biological functions, such as flavonoids, carbohydrates, sterols, quinones, alkaloids and others. Extracts derived from vascular plants are of greatest interest, while data relating to the extracts obtained from mosses are exceedingly scarce. Thus, the data presented in the paper regarding the cytotoxic effect of the tested extracts are unique and difficult to relate to literature data. Klavina et al.^65^ analyzed many species of mosses, including *P*. *schreberi*, with no exact sampling locations given, stating that these included moist coniferous forests, moist deciduous forests and bogs in Latvia. Interestingly, two different extrahents (chloroform, ethanol) were used and both types of the extracts were tested on human and mouse cell lines of cancer origin^65^. The cytotoxicity effect was evaluated with the same method as that used in the presented study (MTT), the extracts were tested at different concentrations and the cells were exposed for 72 h. Aside from the fact that the range of concentrations tested is not explicitly indicated, it is worth noting that none of the extracts (including *P. schreberi*) showed a cytotoxic effect, which is in accordance with our observations (Fig. 1), as only the IV-7 extract (material collected from a site located in a young forest, the age of which was estimated at 5–15 years), and only at the highest concentration (1000 µg/mL) exhibited cytotoxic properties (survival rate below 50%). Interestingly, also none of the 33 methanolic moss extracts examined by Marques et al.^66^ showed a cytotoxic effect against the murine macrophages line RAW264.7 when the cells were treated with 100 µg/ml of extracts for 24 h. Furthermore, among the analyzed extracts, two moss species significantly inhibited LPS-induced NO production without cytotoxic effects^66^. In our model, at a concentration of 125 µg/ml (the most correlative to the one used by Marques et al.^66^, no cytotoxic effect was noted either, what is more the IV-8 extract (obtained from a moss sample collected from a 50-year-old pine forest) seemed to enhance the proliferative properties of L929 cells during 24 h treatment.

Similarly to the results reported by Wolski et al.^1^ regarding the biological activity of the extracts from *Ceratodon purpureus* (Hedw.) Brid., *Dryptodon pulvinatus* (Hedw.) Brid., *Hypnum cupressiformen* Hedw., *Rhytidiadelphus squarossus* (Hedw.) Warnst., and *Tortula muralis* Hedw., extracts from* P*. *schreberi* showed no direct biostatic/biocidal activity against a wide rangeof bacteria and fungi. The only exception was extract no. IV-7 (plants from a *Pinus sylvestris* forest, monoculture up to 15 years), which exhibited fungistatic and fungicidal activity against *C. glabrata* ATCC 90030 (Table 5). The obtained MIC (1000 µg/mL) was similar to that demonstrated for methanol extract of *Plagiochila beddomei* Steph. against *C*. *albicans* (750 µg/mL) by Manoj and Murugan^67^. Moreover, Manoj and Murugan^68^ revealed on a rat model that methanolic and aqueous extracts from *P. beddomei* Steph. supported also wound healing by the intensification of granulation tissue formation, collagen production and angiogenesis. Therefore, we decided to evaluate the pro-regenerative properties of *P*. *schreberi* extracts using an in vitro scratch assay. In contrast, all of the tested *P*. *schreberi* extracts weakened human foreskin fibroblasts line HFF-1 migration and wound healing compared to appropriate controls (Table 6, Figs. 2 and 3). Thus, we did not find pro-regenerative properties of *P*. *schreberi* extracts, and neither the phytocenosisof *P*. *schreberi* nor the composition of the extracts had an effect on it.

The hypothesis established in the present research that the phytocenosis in which a *P*. *schreberi* grow affects the parameters of the extracts, has been partially confirmed. Phytocenosis actually affects some parameters of moss extracts such as antioxidative activity and thus may determine their biological and medical potential.

## Methods

### Object of research and selection of phytocoenoses

One of the most common species of mosses in Poland—*Pleurozium schreberi*—, which is a typical species for all mixed and coniferous forests throughout the Northern Hemisphere^69^ was selected for the study.

**Figure 4:**
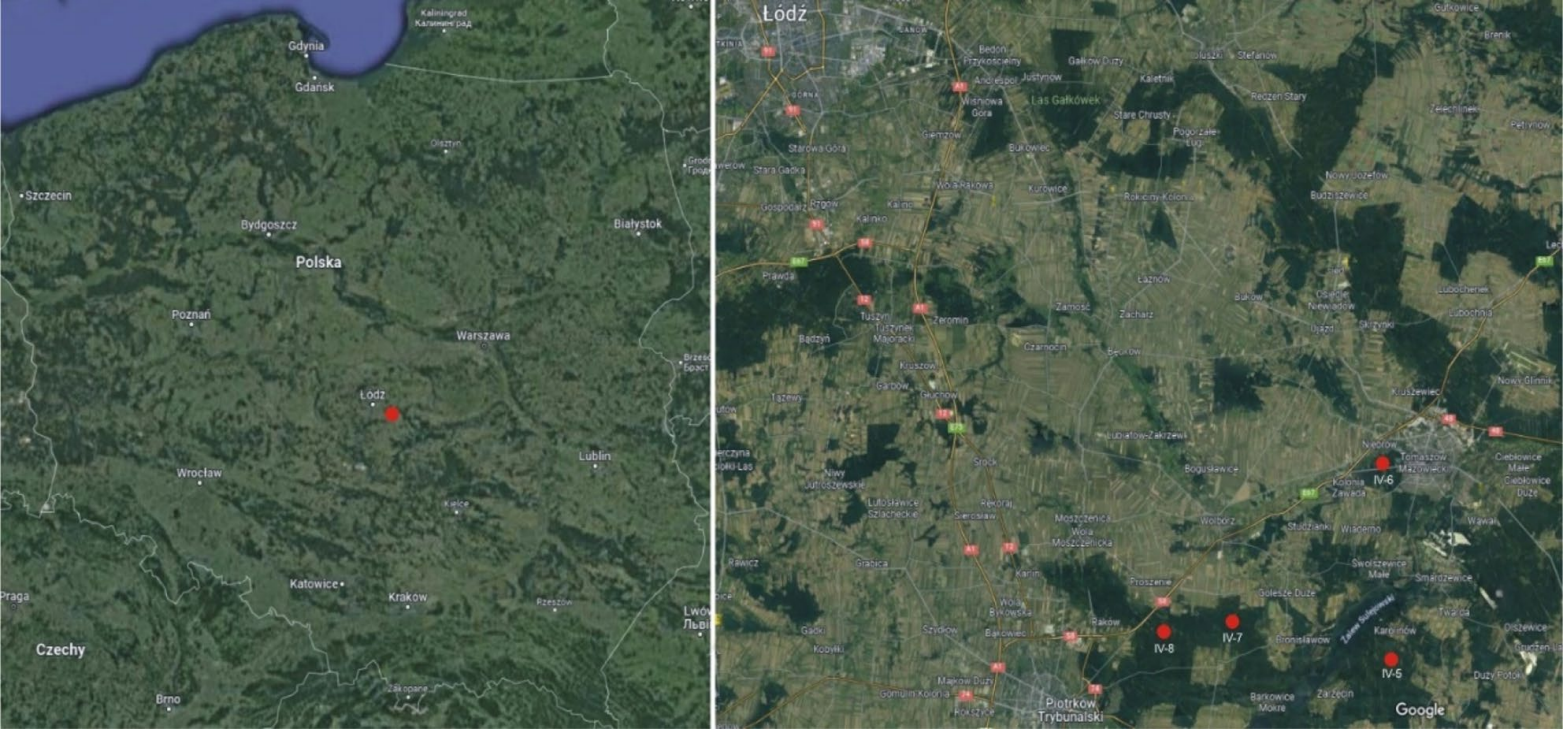
Location of the sampling site—*Pleurozium schreberi* in the Łódź Voivodeship (created by Grzegorz J. Wolski in CorelDRAW 12 software based on Google Maps, free access in June15, 2023).

**Figure 5:**
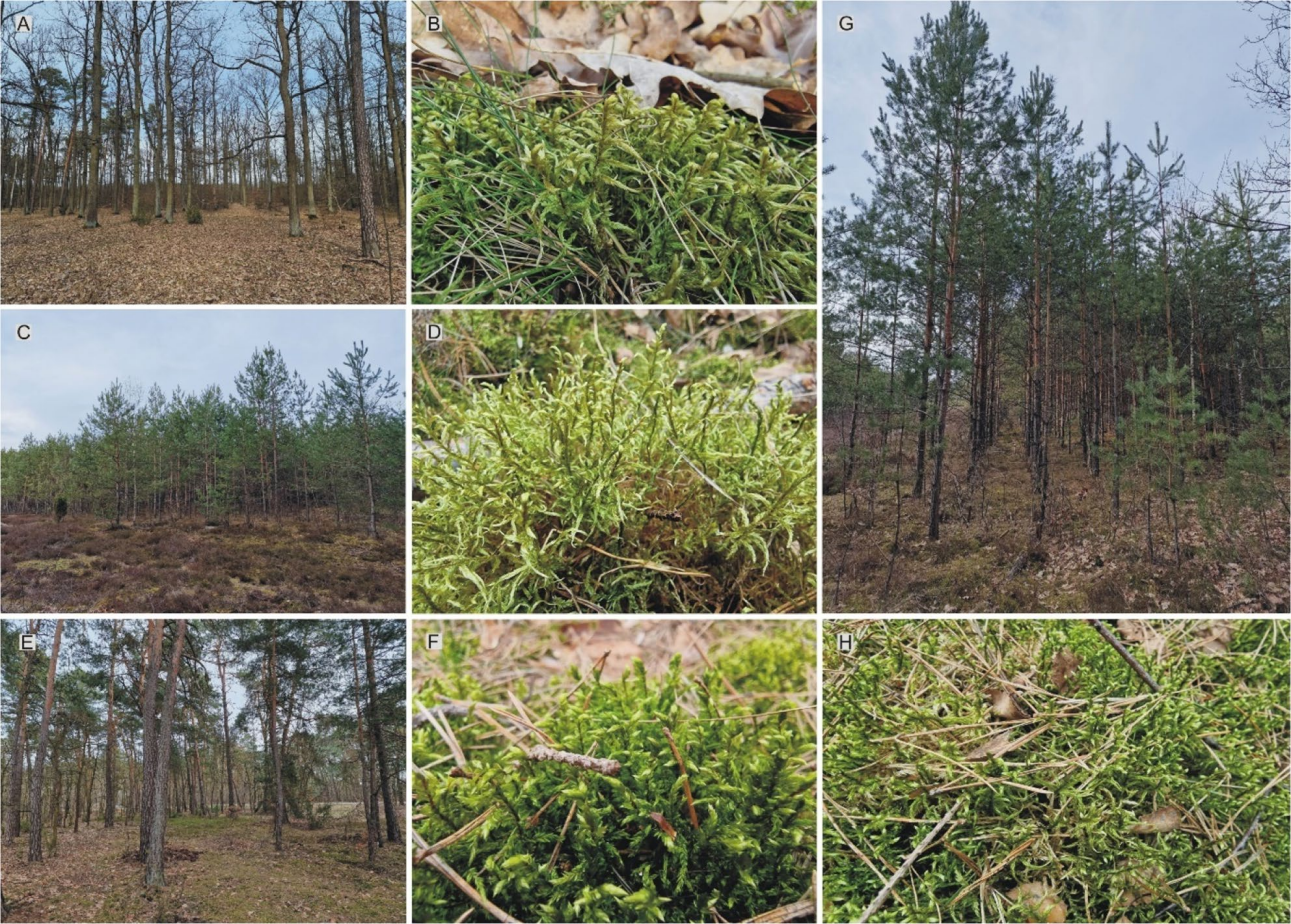
Study of the phytocenosis and turf of the examined species. (**A**,**B**) (phytocenosis IV-5) (**A**) from a mixed forest; (**B**) *P*. *schreberi* from a mixed forest; (**C**,**D**) (phytocenosis IV-6) (**C**) from a forest tract in a pine forest; (**D**) *P*. *schreberi* from a duct in a pine forest; (**E**,**F**) (phytocenosis IV-8) (**E**) from a 50-year-old pine forest; (**F**) *P*. *schreberi* from a 50-year-old pine forest; (**G**,**H**) (phytocenosis IV-7) (**G**) from a 5–15-year-old pine forest; (**H**) *P*. *schreberi* from a 5–15-year-old pine forest (all field photos were taken by Grzegorz J. Wolski).

To achieve the goals set in the manuscript, four different phytocenoses were chosen: IV-5 in a mixed forest; IV-6 in at a forest tract in a pine forest (monoculture); IV-7 in a *Pinus sylvestris* forest (monoculture up to 15 years old) and IV-8 in a *Pinus sylvestris* forest (pine monoculture about 50 years old) (Figs. 4 and 5, and Table 1).The material was collected in spring (March–April) 2022.

Both the plants and the areas from which they were collected are not protected by law. The studied species is very common throughout Europe or even the entire Northern Hemisphere, not covered by any legal protection. All methods were performed in accordance with the relevant guidelines/regulations/national law.

The light measurement tests were performed at the same time, on the same day, during the material selection, using a standard digital illuminance meter (Standard Instruments Co. LTD; AB-1308).

### Extract preparation

The weighed fresh plant material was air-dried at 40˚C until constant mass was reached. Dry samples were ground in a mortar with a pestle to the powder and extracted with pure ethanol at a solid to liquid ratio 1:10 (w/v) for 30 min, at room temperature on the rocker. The supernatant was decanted and the sediment was boiled for 2 min with pure ethanol. The combined ethanol extracts were centrifuged for 10 min at 4500×*g*, 4 °C (3K30, Sigma). After removal of ethanol at ≤ 40 °C with a vacuum rotary evaporator (RV06-ML IKA-WERKE, Germany) the extracts were dissolved in 96% ethanol to the same concentration of 50 mg/mL and stored in the freezer.

### Phenolic content estimation

UPLC/MS analysis was performed on an ultra-performance liquid chromatograph (Waters Acquity UPLC system, Milford, MA, USA) equipped with a binary pump, an autosampler, a column compartment, and a diode array detector. Briefly, the samples were eluted with a gradient of solvent A (4.5% formic acid in ultrapure water) and B (acetonitrile) on an Acquity UPLC HSS T3 C18 column (150 × 2.1 mm, 1.8 μm; Waters) operating at 30 °C, as described in the previous work^74^ with slight modifications. The gradient program was as follows: initial conditions 99% (A), 3 min 75% (A), 10 min 60% (A), 12,5 min 100% (B), 15.0 min 99% (A). The flow rate was 0.45 mL/min and the injection volume was 5 μL. The identification of phenolic compounds by the UPLC-QTOF-MS method had been described in detail previously^74,75^. The mass spectrometer was operating in the negative mode for a mass range of 150–1500 Da, fixed source temperature at 100 °C, desolvation temperature 250 °C, desolvation gas flow 600 L/h, cone voltage 45 V, capillary voltage 2.0 kV, collision energy 50 V. Leucine enkephalin was used as a lock mass. The instrument was controlled by the Mass-LynxTM V 4.1 software. The presence of chlorogenic acid and cryptochlorogenic acid was confirmed by a comparison with authentic standards. The other compounds were tentatively identified on the basis of their UV–Vis spectra, MS, and MS2 properties in comparison with the literature data.

The contents of chlorogenic acid and cryptochlorogenic acid were quantified using corresponding standard calibration curves. A quantitative analysis of the other phenolics was based on the following standards: chlorogenic acid was used for the hydroxycinnamic acid derivatives, and quercetin 3-glucoside for the quercetin derivative. The analysis was repeated three times, and the results were expressed as mg per gram of sample.

Prior to the injection into the UPLC/MS system, the crude ethanolic extracts (6 mL were purified using Sep-Pak Cl8 cartridges (10 g capacity, Waters Corp., Milford, MA). In the first stage, ethanol was completely removed from the extract at 40 °C, using a rotary evaporator (BÜCHI Rotavapor R-114, BÜCHI, Lausanne, Switzerland). The dry residue was dissolved in 3 mL of MilliQ water (Simplicity® Water Purification System, Millipore, Marlborough, MA, USA) and applied to the preconditioned Sep-Pak by sequentially passing 60 mL absolute methanol and 60 mL of MilliQ water through the cartridge. Interfering water-soluble components were removed with water (60 mL) while absorbed phenolics were recovered with methanol (60 mL). The solvent was allowed to evaporate completely under reduced pressure and solid residue was dissolved in 2 mL of methanol and used for UPLC/MS analysis.

Total Phenolic Content estimation was carried out using the Folin-Ciocâlteu’s reagent, according to Singelton and Rossi^70^. Quantification was done on the basis of the standard curve of gallic acid (solution 20–200 μg/mL). The results are expressed as mg gallic acid equivalents (GAE)/g dry extract, mean value of three replicates.

500 μL of the extract (diluted before measurements to the concentration of 2.5 mg/mL) was combined with 3.65 mL of distilled water, 100 μL of the Folin-Ciocâlteu’s reagent and 1000 μL of 10% Na_2_CO_3_. The mixture was vortexed thoroughly and, after incubation at room temperature in darkness for 60 min, the absorbance was measured at 765 nm against a ‘blank’ without the sample extract (UV–Vis spectrophotometer U-2001, Hitachi).

### Assessment of chlorophyll *a* and chlorophyll *b* content

Concentrations of chorophyll *a* and chlorophyll *b* were determined spectrophotometrically in the crude ethanolic extracts according to Miazek and Ledakowicz (2013)^76^. The absorbance (A) of the extract was measured at 648 and 664 nm. Equations for pigments determination (µg/mL of extract) were as follows:

- Chlorophyll *a* = 13.36*A_(664 nm)-5.19*A_648 nm
- Chlorophyll *b* = 27.43*A_(648 nm)-8.12*A_664 nm

The measurements were repeated twice, and chlorophylls amounts were expressed as mg per 1 g of extract.

Statistical analysis was performed using one-way ANOVA followed by Tukey's post hoc test to compare the content of individual phenolic compounds between *P. schreberi* extracts. Statistically significant differences were set at *p* < 0.05.

### Antioxidant evaluation

*Pleurozium schreberi* used in our experiments were collected from IV-5–IV-8 and screened for their antioxidant properties. Due to the fact that different antioxidants have varying reactivity and substrate specificity, two various methods were applied: the ferric reducing antioxidant power (FRAP) and the 2,2′‐azinobis‐3‐ethylbenzotiazoline‐6‐sulfonic acid (ABTS^·+^) assay for the measurement of the total radical scavenging capacity (RawDate 2).

The modified method of Re et al.^71^ was used to determine the antiradical activity. Briefly, a fresh solution of ABTS^·+^ was prepared by dissolving 19.5 mg 2,2′‐azinobis (3‐ethylbenzthiazoline‐6‐sulphonic acid) (ABTS; Sigma, Germany) and 3.3 mg potassium persulphate (dipotassium peroxodisulphate; Sigma, Germany) in 7 mL of 0.1 mol/L phosphate buffer, pH 7.4. This solution was stored in the dark for 12 h for the completion of the reaction. Bryophytes extracts were diluted with deionized water to the concentration of 2.5 mg/mL. 50 μL of *P. schreberi* extract was added to 2 ml ABTS^·+^solution in 0.01 mol/L phosphate buffer, pH 7.4, diluted (usually approximately 1:80) to give an absorbance of about 0.9 and read at 734 nm. The extent of ABTS^·+^decolorization (a decrease in absorbance, corrected for a small decrease in absorbance of ABTS^·+^solution alone) is proportional to the activity of antioxidants in a given sample. Calculations were made on the basis of standard curves obtained for a Trolox solution (range 10–1000 μM). Antioxidant capacity of plant extracts was expressed as the Trolox equivalent [μM].

For the ferric ion reducing antioxidant power (FRAP) assay, a modified method of Benzie and Strain^72^ was used. Working solution was prepared immediately before measurements by mixing 10 volumes of acetate buffer, pH 3.6, with 1 volume of 10 mM/L 2,4,6–tris-2-pyridyl-s-triazine (TPTZ; Sigma) and 1 volume of 20 mM/L FeCl_3_ (Sigma, Germany). Bryophytes extracts were diluted with deionized water to the concentration of 2.5 mg/mL. 50 μL of the samples was mixed with 2 mL of the working solution and incubated at 37 °C. After 5 min, the absorbance of the extracts was read at 593 nm in a spectrophotometer against a reagent blank. The increase in absorbance was proportional to the activity of antioxidants in the extracts. Calculations were made on the basis of standard curves obtained for FeSO_4_ solution (range 100–1500 μM). The ferric reducing ability of the samples measured as antioxidant power was expressed as FeSO_4_ equivalent [μM].

The data in RawDate 1–3 represent the means ± standard deviation (± SD). The data were analysed using STATISTICA v.10.0_MR1_PL (StatSoft) software. One-way analysis of variance (ANOVA) followed by Fisher’s LSD test were performed to find the significant differences at p < 0.005 in each experiment. Statistically significant differences were marked as different small letters on graphs.

### Cytotoxicity (MTT assay)

The culture of adherent mouse L929 fibroblasts (CCL-1, ATCC) was established and carried out in RPMI 1640 medium (Biological Industries, Israel), with the addition of 10% FCS (Biological Industries, Israel) and supplemented with: penicillin–streptomycin-L-glutamine solution (100 U/mL, 100 µg/mL, 0.292 mg/mL, respectively; Gibco, United States), in culture flasks (growth area of 25 cm^2^; TPP, Switzerland). The cells, after their detachment from the growth surface (trypsinization with the 0.25% trypsin-1 mM EDTA solution; Gibco, United Kingdom), were used for 3-(4,5-dimethylthiazol-2-yl)-2,5-diphenyltetrazolium bromide (MTT; Sigma, United States) assay. Briefly, after preparing a cell suspension with the density of 1 × 10^5^/mL, it was portioned by 100 µL into the wells of a 96-well flat bottom tissue culture plate (TPP, Switzerland) and incubated for 24 h (37° C, 5% CO_2_). The culture medium was removed and replaced with a medium containing the specified concentrations of test extracts (100 µL/well). After 24, 48 and 72 h of incubation (under the conditions as above), the medium with the added extracts was removed and replaced for 2 h with the solution of MTT (1 mg/mL; 50 µL/well) in Opti-MEM medium with no phenol red (Gibco, United Kingdom), during which the live cells reduced the MTT salt to formazan crystals. After discarding the media from the cells, the crystals were dissolved in 100 µL of isopropanol. Finally, the absorbance was measured at 570 nm using a spectrophotometer Multiskan EX (LabSystems, Finland).

The viability of the cells was evaluated colorimetrically based on the amount of reduced MTT. Various concentrations of the tested moss extracts, ranging from 1.9 to 1000 µg/mL were added to the L929 fibroblasts. Incubation continued for 24, 48, and 72 h. The concentration of the extracts’ solvent (Et-OH) did not exceed 2,5% and did not disturb fibroblast growth. The cell survival was calculated as follows:

$${\text{Survival rate }}\left( \% \right) \, = \, \left[ {\left( {{\text{As}}{-}{\text{Ab}}} \right)/\left( {{\text{Ac}}{-}{\text{Ab}}} \right)} \right] \, \times { 1}00$$

As—absorbance of test sample (the cells exposed to the extract), Ac—absorbance of negative control (the cells in medium alone), Ab—absorbance of blank control (cell-free samples – background absorption).

The multiplied results (n = 9) were statistically analyzed using the ANOVA statistical method.

### Antimicrobial activity assessment

The reference strains of Gram-positive bacteria: *Staphylococcus aureus* ATCC 29213 (MSSA, methicillin-susceptible *S. aureus*), *Staphylococcus aureus* ATCC 43300 (MRSA, methicillin-resistant *S. aureus*), *Staphylococcus epidermidis* ATCC 12228, *Enterococcus faecalis* ATCC 29212, and Gram-negative bacteria: *Escherichia coli* ATCC 25922, *Pseudomonas aeruginosa* ATCC 25619, as well as the fungi: *Candida albicans* ATCC 10231, *Candida glabrata* ATCC 90030 were used in order to evaluate the biostatic/biocidal activity of the extracts from *P*. *schreberi*. The bacteria were grown on tryptic-soy agar (TSA; BTL, Poland), and yeasts on Sabouraud dextrose agar (SDA; BTL, Poland) for 24 h at 37 °C. Then, microbial suspensions (about 5 × 10^5^ CFU/mL) were prepared in Mueller–Hinton broth (MHB; BTL, Poland) or in RPMI-1640 medium with L-glutamine (Sigma-Aldrich/Merck, Germany) containing 2% glucose (RPMI/Glu) for the bacteria or fungi, respectively. The broth microdilution method was used to determine MIC of *P*. *schreberi* extracts according to the EUCAST guidelines^73^. Initial solutions of the tested extracts (50 mg/mL in 96% ethanol) were diluted in MHB or RPMI/Glu (for the bacteria or fungi, respectively) to the final concentration range of 31.2–2000 µg/mL in 96-well culture plates (Falcon, USA) in the volume of 100 µL/well. Then, microbial suspensions (5 × 10^5^ CFU/mL, 100 µL) were added and the plates were incubated at 37 °C for 24 h (bacteria) or 48 h (fungi). Appropriate microbial growth control was simultaneously prepared: microbial suspensions in medium alone (K1) and microbial suspensions in medium containing 4% ethanol being the highest concentration of the solvent for the extracts in the tested samples (K2). The effect of the selected antibiotics was also tested: oxacillin (Oxa; 0.015–2 µg/mL) against staphylococci, gentamycin (Gen; 0.25–32 µg/mL) against other bacteria, and fluconazole (Flu; 0.5–64 µg/mL) against yeast. MIC was defined as the lowest concentration of the tested extract inhibiting visible bacterial/fungal growth during the co-incubation time compared to the appropriate growth control. MBC/MFC of the extracts referred to the lowest concentration that killed 99.9% of microbial inoculum added to the wells. Thus, there was no bacteria or yeast growth after subculturing 10 µL from the wells marked as MIC or higher concentrations on TSA/SDA (incubation 24–48 h at 37 °C). Experiments were carried out in duplicate for each extract.

### Wound healing (scratch assay)

Pro-regenerative activity of *P*. *schreberi* extracts was tested in vitro using human foreskin fibroblasts (HFF-1 line; LGC Standards, UK). The cells (2.5 × 10^5^ cells/mL) were cultured for 24 h in Dulbecco’s Modified Eagle Medium (DMEM) supplemented with 15% fetal bovine serum (FBS; Biological Industries, USA) and penicillin–streptomycin solution (P/S; Biowest, France) in 24-well plates (Nunc, Denmark) at 37 °C, 5% CO_2_ to obtain a confluent monolayer. The reference lines had been previously marked on the bottom of the wells. Then, the cell monolayers were gently scratched across the reference lines to form wounds and detached cells were removed. The photos of the prepared wounds were immediately taken (T0) using the Motic Microscope model AE-2000 T with an inverted field of view and integrated camera (Conbest, Poland). Finally, fresh culture medium alone (control K1—wound healing in medium), culture medium with 1% ethanol (control K2—wound healing in the presence of the solvent for the extracts at the highest concentration obtained in the tested samples) or culture medium with *P*. *schreberi* extracts used at the concentrations 100 and 500 µg/mL were added. The cells were exposed to the extracts for 24 h, after which the wounds were photographed again (T24). The obtained images were analyzed using the ImageJ software to compare total wound areas before and after cell exposition. The percentage of wound closure was calculated according to the following formula: [(wound area T0–wound area T24)/wound area T0] × 100. The experiment was carried out twice.

### Supplementary Information

Supplementary Information.

## Data Availability

All data generated or analyzed during this study are included in this published article [and its supplementary information files].
