# Supplementary material for: Influence of phytocenosis on the medical potential of moss extracts: the Pleurozium schreberi (Willd. ex Brid.) Mitt. case
Source: Sci Rep. 2023 Nov 20;13:20293. doi: 10.1038/s41598-023-47654-z (PMC10661538; doi:10.1038/s41598-023-47654-z)
Supplement: Supplementary file 1 — Supplementary Information. [file 41598_2023_47654_MOESM1_ESM.docx]

**Raw Data 1**


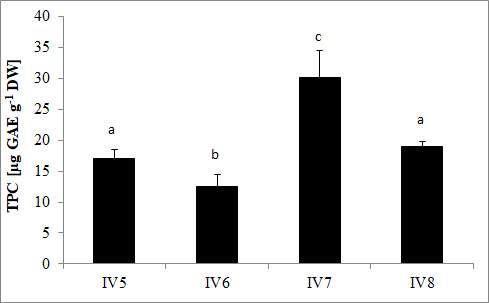


**Supplementary figure 1.** The total phenolic content of selected mosses expressed as µg gallic acid equivalent per 1g of dry extract. The results are expressed as mean values of 3 independent experiments ± SD. Statistically different values are shown by dissimilar letters (*p*≤0.005; ANOVA variance analysis followed by Fisher’s LSD test).

**Raw Data 2**


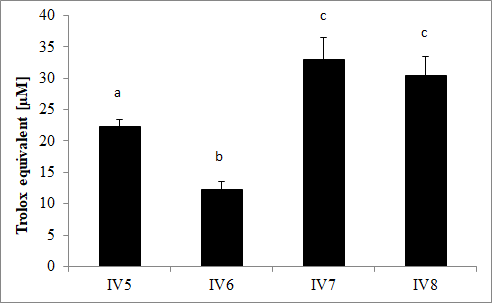


**Supplementary figure 2.** The ABTS cation scavenging activity of selected mosses extracts calculated as Trolox equivalent [µM]. The results are expressed as mean values of 3 independent experiments ±SD. Statistically different values are shown by dissimilar letters (*p*≤0.005; ANOVA variance analysis followed by Fisher’s LSD test).

**Raw Data 3**

**
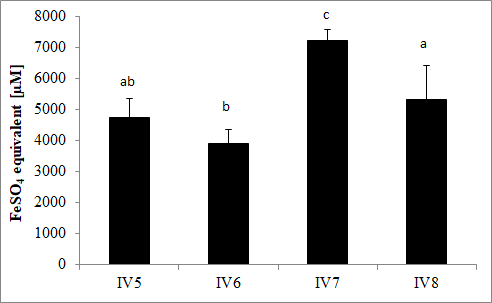
**

**Supplementary figure 3.** Reducing antioxidant power of mosses extracts calculated as FeSO4 equivalent [µM]. The results are expressed as mean values of 3 independent experiments ±SD. Statistically different values are shown by dissimilar letters (*p*≤0.005; ANOVA variance analysis followed by Fisher’s LSD test).
